# Supplementary material for: Cost-Effectiveness of Therapeutic Drug Monitoring of Anti-TNF Therapy in Inflammatory Bowel Disease: A Systematic Review
Source: Pharmaceutics. 2022 May 7;14(5):1009. doi: 10.3390/pharmaceutics14051009 (PMC9145467; doi:10.3390/pharmaceutics14051009)
Supplement: Supplementary file 1 [file pharmaceutics-14-01009-s001.zip › pharmaceutics-1681814-supplementary.pdf]

Supplementary material

Cost-Effectiveness of Therapeutic Drug Monitoring of Anti-TNF Therapy in Inflammatory Bowel Disease: A Systematic Review

Silvia Marquez-Megias [1,†], Ricardo Nalda-Molina [1,2,*,†], Javier Sanz-Valero [3,4], Patricio Más-Serrano [1,2,5], Marcos Diaz-Gonzalez [2], Maria Remedios Candela-Boix [6] and Amelia Ramon-Lopez [1,2]

[1] School of Pharmacy, Miguel Hernández University, 03550 San Juan de Alicante, Spain; silvia.marquez@goumh.umh.es (S.M.-M.); mas_pat@gva.es (P.M.-S.); aramon@umh.es (A.R.-L.)
[2] Alicante Institute for Health and Biomedical Research (ISABIAL-FISABIO Foundation), 03010 Alicante, Spain; diaz_marcosgon@gva.es
[3] Department of Public Health and History of Science, School of Medicine, Miguel Hernandez University, 03550 Alicante, Spain; fj.sanz@isciii.es
[4] Carlos III Health Institute, National School of Occupational Medicine, 28029 Madrid, Spain
[5] Clinical Pharmacokinetics Unit, Pharmacy Department, Alicante University General Hospital, 03010 Alicante, Spain
[6] Virgen de la Salud General Hospital of Elda, 03600 Elda, Spain; candela_marboi@gva.es
* Correspondence: jnalda@umh.es
† These authors contributed equally to this work.

Citation: Marquez-Megias, S.; Nalda-Molina, R.; Sanz-Valero, J.; Más-Serrano, P.; Diaz-Gonzalez, M.; Candela-Boix, M.R.; Ramon-Lopez, A. Cost-Effectiveness of Therapeutic Drug Monitoring of Anti-TNF Therapy in Inflammatory Bowel Disease: A Systematic Review. *Pharmaceutics* **2022**, *14*, 1009. https://doi.org/10.3390/pharmaceutics14051009

Academic Editor: Koyo Nishida

Received: 30 March 2022
Accepted: 30 April 2022
Published: 7 May 2022

Publisher's Note: MDPI stays neutral with regard to jurisdictional claims in published maps and institutional affiliations.

Copyright: © 2022 by the authors. Licensee MDPI, Basel, Switzerland. This article is an open access article distributed under the terms and conditions of the Creative Commons Attribution (CC BY) license (https://creativecommons.org/licenses/by/4.0/).

Supplementary material

Table S1. PRISMA 2020 Checklist
Table S2. Search strategies
Table S3. List of excluded studies and reasons
Table S4. CHEERS checklist assessment

Table S1. PRISMA 2020 Checklist

Section and Topic	Item #	Checklist item	Location where ítem is reported
**TITLE**			
Title	1	Identify the report as a systematic review.	Title page
**ABSTRACT**			
Structured summary	2	Provide a structured summary including, as applicable: background; objectives; data sources; study eligibility criteria, participants, and interventions; study appraisal and synthesis methods; results; limitations; conclusions and implications of key findings; systematic review registration number.	Abstract, Title page
**INTRODUCTION**			
Rationale	3	Describe the rationale for the review in the context of existing knowledge.	Introduction: paragraph 1-4
Objectives	4	Provide an explicit statement of the objective or question the review addresses.	Introduction: paragraph 5
**METHODS**			
Eligible criteria	5	Specify the inclusión and exclusión criteria for the review and how studies were grouped for the syntheses	Methods 2.5
Information sources	6	Specify all databases, registers, websites, organizations, reference lists and other sources searched or consulted to identify studies. Specify the date when each source was last searched or consulted.	Methods 2.2. 2.4 paragraph 4
Search strategy	7	Present the full search strategies for all databases, registers and websites, including any filters and limits used.	Supplementar y Table S2
Selection process	8	Specify the methods used to decide whether a study met the inclusion criteria of the review, including how many reviewers screened each record and each report retrieved, whether they worked independently, and if applicable, details of automation tools used in the process.	Methods 2.5
Data collection process	9	Specify the methods used to collect data from reports, including how many reviewers collected data from each report, whether they worked independently, any processes for obtaining or confirming data from study investigators, and if applicable, details of automation tools used in the process.	Methods 2.7
Data ítems	10	List and define all other variables for which data were sought (e.g. participant and intervention characteristics, funding sources). Describe any assumptions made about any missing or unclear information.	Methods 2.7
Study risk of bias assessment	11	Specify the methods used to assess risk of bias in the included studies, including details of the tool(s) used, how many reviewers assessed each study and whether they worked independently, and if applicable, details of automation tools used in the process.	Methods 2.5 Paragraph 4
Effect measures	12	Specify for each outcome the effect measure(s) (e.g. risk ratio, mean difference) used in the synthesis or presentation of results	Methods 2.7

Syntesis methods	13	Describe the processes used to decide which studies were eligible for each synthesis (e.g. tabulating the study intervention characteristics and comparing against the planned groups for each synthesis (item #5)).	Methods 2.5
Reporting bias assessment	14	Describe any methods used to assess risk of bias due to missing results in a synthesis (arising from reporting biases).	Methods 2.6 paragraph 3
Certainty assessment	15	Describe any methods used to assess certainty (or confidence) in the body of evidence for an outcome.	Methods 2.6 paragraph 3
**RESULTS**			
Study selection	16	Describe the results of the search and selection process, from the number of records identified in the search to the number of studies included in the review, ideally using a flow diagram. Cite studies that might appear to meet the inclusion criteria, but which were excluded, and explain why they were excluded.	Figure 1 Supplementar y table S3
Study characteristics	17	Cite each included study and present its characteristics.	Table 1 Results 3.1, 3.2, 3.3, 3.4, 3.5, 3.6
Risk of bias in studies	18	Present assessments of risk of bias for each included study.	Results Paragraph 1
Results of individual studies	19	For all outcomes, present, for each study: (a) summary statistics for each group (where appropriate) and (b) an effect estimate and its precision (e.g. confidence/credible interval), ideally using structured tables or plots.	Table 1
Results of syntheses	20	For each synthesis, briefly summarize the characteristics and risk of bias among contributing studies.	NA
Reporting biases	21	Present assessments of risk of bias due to missing results (arising from reporting biases) for each synthesis assessed.	Results 3.6
Certainty of evidence	22	Present assessments of certainty (or confidence) in the body of evidence for each outcome assessed.	Results 3.6 paragraph 3
**DISCUSSION**			
Discussion	23	Provide a general interpretation of the results in the context of other evidence. Discuss any limitations of the evidence included in the review. Discuss any limitations of the review processes used. Discuss implications of the results for practice, policy, and future research	Discussion pages
**OTHER INFORMATION**			
Registration and protocol	24	Provide registration information for the review, including register name and registration number, or state that the review was not registered.	Methods 2.4
Support	25	Describe sources of financial or non-financial support for the review, and the role of the funders or sponsors in the review.	Declarations Funding
Competing interests	26	Declare any competing interests of review authors.	Declarations
Availability of data, code and other materials	27	Report which of the following are publicly available and where they can be found: template data collection forms; data extracted from included studies; data used for all analyses; analytic code; any other materials used in the review.	Declarations

NA: Not applicable

Table S2. Search strategies for each data base.

Database	Search strategy
Medline (via PubMed)	• Target population (Inflammatory bowel diseases)<br>"Inflammatory Bowel Diseases"[Mesh] OR "Inflammatory Bowel Disease*"[Title/Abstract] OR "Idiopathic Proctocolitis"[Title/Abstract] OR "Ulcerative Colitis"[Title/Abstract] OR "Colitis Gravis"[Title/Abstract] OR "Colitis Ulcerativa"[Title/Abstract] OR "Colitis Ulcerosa"[Title/Abstract] OR "Histiocytic Ulcerative Colitis"[Title/Abstract] OR "Mucosal Colitis"[Title/Abstract] OR "Ulcerative Colorectitis"[Title/Abstract] OR "Ulcerative Proctocolitis"[Title/Abstract] OR "Ulcerous Colitis"[Title/Abstract] OR "Crohn's Enteritis"[Title/Abstract] OR "Regional Enteritis"[Title/Abstract] OR "Crohns Disease"[Title/Abstract] OR "Crohn's Disease"[Title/Abstract] OR "Granulomatous Enteritis"[Title/Abstract] OR "Ileocolitis"[Title/Abstract] OR "Granulomatous Colitis"[Title/Abstract] OR "Terminal Ileitis"[Title/Abstract] OR "Regional Ileitis"[Title/Abstract] OR "Enteritis Regionalis"[Title/Abstract] OR "Morbus Crohn*"[Title/Abstract] OR "Regional Enterocolitis"[Title/Abstract]<br>• Intervention (Tumor necrosis factor inhibitors)<br>"Tumor Necrosis Factor Inhibitors"[Mesh] OR "Tumor Necrosis Factor Inhibitor*"[Title/Abstract] OR "Tumor Necrosis Factor Blocker*"[Title/Abstract] OR "TNF Inhibitor*"[Title/Abstract] OR "TNF Blocker*"[Title/Abstract] OR "Tumor Necrosis Factor Antagonist*"[Title/Abstract] OR "TNF Antagonist*"[Title/Abstract] OR "TNF Inhibitor*"[Title/Abstract] OR "Tumor Necrosis Factor-a"[Title/Abstract] OR "TNF-a" [Title/Abstract] OR "anti TNF"[Title/Abstract] OR "TNF Alpha Inhibitor*"[Title/Abstract] OR "Anti Tumor Necrosis Factor Agent*"[Title/Abstract] OR "Tumour Necrosis Factor Alpha Inhibitor*"[Title/Abstract] OR "Adalimumab"[Mesh] OR "Adalimumab"[Title/Abstract] OR "Humira"[Title/Abstract] OR "Adalimumab-adbm"[Title/Abstract] OR "Amjevita"[Title/Abstract] OR "Adalimumab-atto"[Title/Abstract] OR "Cyltezo"[Title/Abstract] OR "Infliximab"[Mesh] OR "Infliximab"[Title/Abstract] OR "Monoclonal Antibody cA2"[Title/Abstract] OR "MAb cA2"[Title/Abstract] OR "Infliximab-abda"[Title/Abstract] OR "Renflexis"[Title/Abstract] OR "Infliximab-dyyb"[Title/Abstract] OR "Inflectra"[Title/Abstract] OR "Remicade"[Title/Abstract]<br>• Results (Cost effectiveness of drug monitoring)

"Cost-Benefit Analysis"[Mesh] OR "Cost-Benefit Analysis"[Title/Abstract] OR "Cost Benefit Analyses"[Title/Abstract] OR "Cost Effectiveness"[Title/Abstract] OR "Cost Benefit Data"[Title/Abstract] OR "Cost Utility Analysis"[Title/Abstract] OR "Cost-Utility Analyses"[Title/Abstract] OR "Economic Evaluation*"[Title/Abstract] OR "Marginal Analysis"[Title/Abstract] OR "Marginal Analyses"[Title/Abstract] OR "Cost Benefit"[Title/Abstract] OR "Costs and Benefit*"[Title/Abstract] OR "Benefits and Cost*"[Title/Abstract] OR "Cost Effectiveness Analysis"[Title/Abstract] OR "Cost Analysis"[Title/Abstract] OR "Cost Benefit"[Title/Abstract] OR "Cost Benefit Ratio"[Title/Abstract] OR "Cost Savings"[Mesh] OR "Cost Saving*"[Title/Abstract] OR "Cost Audit"[Title/Abstract] OR "Cost Containment"[Title/Abstract] OR "Cost Control"[Title/Abstract] AND "Drug Monitoring"[Mesh] OR "Drug Monitoring"[Title/Abstract] OR "Therapeutic Drug Monitoring"[Title/Abstract] OR "Medication Monitoring"[Title/Abstract]

The final search equation was developed for use in MEDLINE via PubMed through the Boolean union of the 3 proposed equations (Population AND Intervention AND Result).

Embase

- Target population (Inflammatory bowel diseases)

('inflammatory bowel disease'/exp OR 'inflammatory bowel disease' OR 'inflammatory bowel diseases' OR 'crohn disease'/exp OR 'crohn disease' OR 'crohn's disease' OR 'crohns disease' OR 'cleron disease' OR 'enteritis regionalis' OR 'intestinal tract, regional enteritis' OR 'morbus crohn' OR 'regional enteritis' OR 'regional enterocolitis' OR 'ulcerative colitis'/exp OR 'chronic ulcerative colitis' OR 'colitis ulcerativa' OR 'colitis ulcerosa' OR 'colitis ulcerosa chronica' OR 'colitis, mucosal' OR 'colitis, ulcerative' OR 'colitis, ulcerous' OR 'colon, chronic ulceration' OR 'histiocytic ulcerative colitis' OR 'mucosal colitis' OR 'ulcerative colitis' OR 'ulcerative colorectitis' OR 'ulcerative procto colitis' OR 'ulcerative proctocolitis' OR 'ulcerous colitis')

- Intervention (Tumor necrosis factor inhibitors)

('tumor necrosis factor inhibitor'/exp OR 'tnf alpha inhibitor' OR 'tnf inhibitor' OR 'anti tnf agent' OR 'anti tnf alpha agent' OR 'anti tumor necrosis factor agent' OR 'anti tumour necrosis factor agent' OR 'tumor necrosis factor alpha inhibitor' OR 'tumor necrosis factor inhibitor' OR 'tumor necrosis factor inhibitors' OR 'tumour necrosis factor alpha inhibitor' OR 'tumour necrosis factor inhibitor' OR 'adalimumab'/exp OR 'abp 501' OR 'abp501' OR 'abrilada' OR 'abt d2e7' OR 'abtd2e7' OR 'adalimumab' OR 'adalimumab adaz' OR 'adalimumab adbm' OR 'adalimumab afzb' OR 'adalimumab atto' OR 'adalimumab beta' OR 'adalimumab bwwd' OR 'adalimumab fkjp' OR 'adalimumab-adaz' OR 'adalimumab-adbm' OR 'adalimumab-afzb' OR 'adalimumab-atto' OR 'adalimumab-bwwd' OR 'adalimumab-fkjp' OR 'adaly' OR 'amgevita' OR 'amjevita' OR 'amsparity' OR 'avt 02' OR 'avt02' OR 'bat 1406' OR 'bat1406' OR 'bax 2923' OR 'bax 923' OR 'bax2923' OR 'bax923' OR 'bcd 057' OR 'bcd057' OR 'bi 695501' OR 'bi695501' OR 'chs 1420' OR 'chs1420' OR 'cinnora' OR 'ct p17' OR 'ctp17' OR 'cyltezo' OR 'da 3113' OR

	'da3113' OR 'dmb 3113' OR 'dmb3113' OR 'exemptia' OR 'fkb 327' OR 'fkb327' OR 'fyzoclad' OR 'gp 2017' OR 'gp2017' OR 'hadlima' OR 'halimatoz' OR 'hefiya' OR 'hlx 03' OR 'hlx03' OR 'hulio' OR 'humira' OR 'hyrimoz' OR 'ibi 303' OR 'ibi303' OR 'idacio' OR 'imraldi' OR 'kromeya' OR 'lu 200134' OR 'lu200134' OR 'm 923' OR 'm923' OR 'mabura' OR 'monoclonal antibody d2e7' OR 'msb 11022' OR 'msb11022' OR 'ons 3010' OR 'ons3010' OR 'pf 06410293' OR 'pf 6410293' OR 'pf06410293' OR 'pf6410293' OR 'qletli' OR 'raheara' OR 'sb 5' OR 'sb5' OR 'solymbic' OR 'trudexa' OR 'yuflyma' OR 'zrc 3197' OR 'zrc3197' OR 'infliximab'/exp OR 'abp 710' OR 'abp710' OR 'avakine' OR 'avsola' OR 'flixabi' OR 'gp 1111' OR 'gp1111' OR 'inflectra' OR 'infliximab' OR 'infliximab abda' OR 'infliximab axxq' OR 'infliximab dyyb' OR 'infliximab qbtx' OR 'infliximab-abda' OR 'infliximab-axxq' OR 'infliximab-dyyb' OR 'infliximab-qbtx' OR 'ixifi' OR 'pf 06438179' OR 'pf 6438179' OR 'pf06438179' OR 'pf6438179' OR 'remicade' OR 'remsima' OR 'renflexis' OR 'revellex' OR 'ta 650' OR 'ta650' OR 'zessly')<br><br>• Results (Cost effectiveness of drug monitoring)<br><br>('cost benefit analysis'/exp OR 'cost analysis' OR 'cost benefit' OR 'cost benefit analysis' OR 'cost benefit ratio' OR 'cost-benefit analysis') AND ('drug monitoring'/exp OR 'drug monitoring' OR 'medication monitoring' OR 'monitoring, drug' OR 'therapeutic drug monitoring')<br><br>The final search equation was developed for use in EMBASE through the Boolean union of the 3 proposed equations (Population AND Intervention AND Result).
Cochrane Library	#1 MeSH descriptor: [Inflammatory Bowel Diseases] explode all trees<br>#2 ("Inflammatory Bowel Diseases"):ti,ab,kw (Word variations have been searched)<br>#3 MeSH descriptor: [Tumor Necrosis Factor Inhibitors] explode all trees<br>#4 ("Tumor Necrosis Factor Inhibitors"):ti,ab,kw (Word variations have been searched)<br>#5 MeSH descriptor: [Cost-Benefit Analysis] explode all trees<br>#6 ("Cost-Benefit Analysis"):ti,ab,kw (Word variations have been searched)<br>#7 MeSH descriptor: [Drug Monitoring] explode all trees<br>#8 ("Drug Monitoring"):ti,ab,kw (Word variations have been searched)<br><br>#9 (#1 OR #2) AND (#3 OR #4) AND ((#5 OR #6) AND (#7 OR #8))
PsycINFO	S1 MA inflammatory bowel disease OR TI inflamatory bowel disease OR AB inflamatory bowel disease<br>S2 MA tumor necrosis factor inhibitors OR TI tumor necrosis factor inhibitors OR AB tumor necrosis factor inhibitors<br>S3 MA cost benefit analysis OR TI cost benefit analysis OR AB cost benefit analysis<br>S4 MA drug monitoring OR TI drug monitoring OR AB drug monitoring<br><br>S5 S1 AND S2 AND (S3 AND S4)
Scopus	• Target population (Inflammatory bowel diseases)

TITLE-ABS-KEY("Inflammatory Bowel Diseases" OR "Inflammatory Bowel Disease" OR "Idiopathic Proctocolitis" OR "Ulcerative Colitis" OR "Colitis Gravis" OR "Colitis Ulcerativa" OR "Colitis Ulcerosa" OR "Histiocytic Ulcerative Colitis" OR "Mucosal Colitis" OR "Ulcerative Colorectitis" OR "Ulcerative Proctocolitis" OR "Ulcerous Colitis" OR "Crohns Enteritis" OR "Regional Enteritis" OR "Crohns Disease" OR "Crohn's Disease" OR "Granulomatous Enteritis" OR "Ileocolitis" OR "Granulomatous Colitis" OR "Terminal Ileitis" OR "Regional Ileitis" OR "Enteritis Regionalis" OR "Morbus Crohn" OR "Regional Enterocolitis")

- Intervention (Tumor necrosis factor inhibitors)

TITLE-ABS-KEY("Tumor Necrosis Factor Inhibitor" OR "Tumor Necrosis Factor Blocker" OR "TNF Inhibitor" OR "TNF Blocker" OR "Tumor Necrosis Factor Antagonist" OR "TNF Antagonist" OR "TNF Inhibitor" OR "Tumor Necrosis Factor-a" OR "TNF-a" OR "anti TNF" OR "TNF Alpha Inhibitor" OR "Anti Tumor Necrosis Factor Agent" OR "Tumour Necrosis Factor Alpha Inhibitor" OR "Adalimumab" OR "Humira" OR "Adalimumab-adbm" OR "Amjevita" OR "Adalimumab-atto" OR "Cyltezo" OR "Infliximab" OR "Monoclonal Antibody cA2" OR "MAb cA2" OR "Infliximab-abda" OR "Renflexis" OR "Infliximab-dyyb" OR "Inflectra" OR "Remicade")

- Results (Cost effectiveness of drug monitoring)

TITLE-ABS-KEY("Cost-Benefit Analysis" OR "Cost Benefit Analyses" OR "Cost Effectiveness" OR "Cost Benefit Data" OR "Cost Utility Analysis" OR "Cost-Utility Analyses" OR "Economic Evaluation" OR "Marginal Analysis" OR "Marginal Analyses" OR "Cost Benefit" OR "Costs and Benefit" OR "Benefits and Cost" OR "Cost Effectiveness Analysis" OR "Cost Analysis" OR "Cost Benefit Ratio" OR "Cost Saving" OR "Cost Audit" OR "Cost Containment" OR "Cost Control") AND TITLE-ABS-KEY("Drug Monitoring" OR "Therapeutic Drug Monitoring" OR "Medication Monitoring")

The final search equation was developed for use in SCOPUS through the Boolean union of the 3 proposed equations (Population AND Intervention AND Result).

Web of Science

- Target population (Inflammatory bowel diseases)

"Inflammatory Bowel Diseases" OR "Inflammatory Bowel Disease" OR "Idiopathic Proctocolitis" OR "Ulcerative Colitis" OR "Colitis Gravis" OR "Colitis Ulcerativa" OR "Colitis Ulcerosa" OR "Histiocytic Ulcerative Colitis" OR "Mucosal Colitis" OR "Ulcerative Colorectitis" OR "Ulcerative Proctocolitis" OR "Ulcerous Colitis" OR "Crohns Enteritis" OR "Regional Enteritis" OR "Crohns Disease" OR "Crohn's Disease" OR "Granulomatous Enteritis" OR "Ileocolitis" OR "Granulomatous Colitis" OR "Terminal Ileitis" OR "Regional Ileitis" OR "Enteritis Regionalis" OR "Morbus Crohn" OR "Regional Enterocolitis" (Topic)

- Intervention (Tumor necrosis factor inhibitors)

"Tumor Necrosis Factor Inhibitor" OR "Tumor Necrosis Factor Blocker" OR "TNF Inhibitor"

	OR "TNF Blocker" OR "Tumor Necrosis Factor Antagonist" OR "TNF Antagonist" OR "TNF Inhibitor" OR "Tumor Necrosis Factor-a" OR "TNF-a" OR "anti TNF" OR "TNF Alpha Inhibitor" OR "Anti Tumor Necrosis Factor Agent" OR "Tumour Necrosis Factor Alpha Inhibitor" OR "Adalimumab" OR "Humira" OR "Adalimumab-adbm" OR "Amjevita" OR "Adalimumab-atto" OR "Cyltezo" OR "Infliximab" OR "Monoclonal Antibody cA2" OR "MAb cA2" OR "Infliximab-abda" OR "Renflexis" OR "Infliximab-dyyb" OR "Inflectra" OR "Remicade" (Topic)<br>● Results (Cost effectiveness of drug monitoring)<br>"Cost-Benefit Analysis" OR "Cost Benefit Analyses" OR "Cost Effectiveness" OR "Cost Benefit Data" OR "Cost Utility Analysis" OR "Cost-Utility Analyses" OR "Economic Evaluation" OR "Marginal Analysis" OR "Marginal Analyses" OR "Cost Benefit" OR "Costs and Benefit" OR "Benefits and Cost" OR "Cost Effectiveness Analysis" OR "Cost Analysis" OR "Cost Benefit Ratio" OR "Cost Saving" OR "Cost Audit" OR "Cost Containment" OR "Cost Control" (Topic) and "Drug Monitoring" OR "Therapeutic Drug Monitoring" OR "Medication Monitoring" (Topic)<br>The final search equation was developed for use in WEB OF SCIENCE through the Boolean union of the 3 proposed equations (Population AND Intervention AND Result).
LILACS	(Inflammatory Bowel Diseases [Subject descriptor] or Inflammatory Bowel Diseases [Words]) and (Tumor Necrosis Factor Inhibitor [Subject descriptor] or Tumor Necrosis Factor Inhibitor [Words]) and (Cost-Benefit Analysis [Subject descriptor] or Cost-Benefit Analysis [Words]) and (Drug Monitoring [Subject descriptor] or Drug Monitoring [Words])
MEDES	("Enfermedades Inflamatorias del Intestino"[título/resumen/palabras_clave]) AND ("Inhibidores del factor de Necrosis Tumorales"[título/resumen/palabras_clave]) AND ("Cost-Benefit Analysis "[título/resumen/palabras_clave]) AND ("Drug Monitoring"[título/resumen/palabras_clave])

Table S3. List of excluded studies and reasons

Reason of exclusion	Excluded studies
Not original article	Bhattacharya A, et al [64]; Alsound D, et al[65]; Fobelo MJ, et al [66]; Felice C, et al [67]; Peyrin-Biroulet L, et al [68]; Martelli L, et al [27]; Mitrev N, et al [69]; Franco DL, et al [70]; Hoseyni H, et al [71]; Lega S, et al [72]; Papamichael K, et al [73]; Teml A, et al [74]; Khanna R, et al [75]; Ricciuto A, et al [76]; Yao J, et al [29]; Yanai H, et al [77]; McNeill RP, et al [78]; Papamichael K, et al [79]; Andrews JM, et al [80]; Tavis S [81]; Rentsch CA et al [82]; Rentsch C et al [83]; Rentsch C et al [84]; Kozak J et al [85]; Janko N, et al [86]; Wright EK et al [87]; Steenholdt C et al [88]; Lee JM, et al [89]; Doherty J, et al [90]; Steen J, et al [91]; Yang SK [92]; Mehta P, et al [93]; Sanchez-Hernandez JG, et al [94]; Zandvliet ML, et al [95]
Causality criterion	Patel RN, et al [96]; Syversen SW, et al [97]; Drobne D, et al [98]; Selinger CP, et al [99]; Thomas PWA, et al [100]; Grossberg LB, et al [101]; Nigam GB, et al [102]; Langford T, et al [103]; Crane H, et al [104]; Stein BN, et al [105]; Sparrow M [106]; Park KT, et al [107]; Kelly OB, et al [108]; Scharnhorst V, et al [109]; Jourdil JF, et al [110]

Table S4. CHEERS checklist assessment.

		Yao et al	Wu et al	Ganesana-nthan et al	Negoescu et al	Attar et al	Guidi et al	Taks et al	Roblin et al	Freeman et al	Vande Casteele et al	Steenholdt et al	Steenholdt et al	Velayos et al
1.	Title	Y	Y	Y	Y	Y	N	N	Y	Y	N	Y	Y	Y
2.	Abstract	Y	Y	N	Y	N	Y	N	N	Y	Y	Y	Y	Y
3.	Background & Objectives	Y	Y	Y	Y	Y	Y	Y	Y	Y	Y	Y	Y	Y
4.	Target Population & Subgroups	Y	Y	Y	Y	N	Y	N	N	Y	Y	Y	Y	Y
5.	Setting & Location	Y	Y	Y	N	N	Y	Y	N	Y	Y	N	N	Y
6.	Study Perspective	Y	Y	N	Y	N	N	N	N	N	Y	Y	Y	N
7.	Comparators	Y	Y	N	Y	Y	Y	N	Y	Y	Y	Y	Y	Y
8.	Time Horizon	N	N	N	N	N	N	N	Y	Y	N	N	N	Y
9.	Discount Rate	Y	N	N	Y	N	N	N	N	Y	Y	N	N	N
10.	Choice of Health Outcomes	Y	N	Y	Y	N	Y	N	N	Y	Y	Y	Y	Y
11.	Measurement of Effectiveness	Y	N	N	N	N	N	N	Y	Y	Y	N	N	N
12.	Measurement & Valuation of Preference-based Outcomes	Y	Y	N	N	N	Y	Y	N	Y	Y	Y	Y	N
13.	Estimating Resources & Costs	Y	N	N	Y	N	Y	N	N	Y	N	Y	Y	Y
14.	Currency, Price Date & Conver-sion	Y	N	N	Y	N	N	N	N	Y	Y	Y	Y	N
15.	Choice of Model	Y	Y	N	Y	Y	Y	N	Y	Y	N	N	N	Y
16.	Assumptions	Y	N	N	Y	N	Y	N	N	Y	Y	Y	Y	Y
17.	Analytic Methods	Y	Y	N	Y	N	Y	N	N	Y	Y	Y	Y	Y
18.	Study Parameters	Y	Y	N	Y	N	Y	N	N	Y	Y	Y	Y	Y
19.	Incremental Costs & Outcomes	Y	N	N	Y	N	Y	N	N	Y	Y	Y	Y	Y
20.	Characterising Uncertainty	Y	N	N	Y	N	N	N	Y	Y	N	N	N	N
21.	Characterising Heterogeneity	Y	N	N	N	N	Y	N	N	Y	N	N	N	N
22.	Study findings, limitations, gen-eralisability and current knowledge	Y	Y	Y	Y	Y	Y	N	Y	Y	Y	Y	Y	Y
23.	Source of funding	N	N	N	Y	Y	Y	N	Y	Y	Y	Y	Y	Y

24.	Conflicts of Interest	N	N	Y	Y	Y	Y	Y	Y	Y	Y	Y	Y	Y
	Total (%)	21 (87.5%)	12 (50%)	7 (29.2%)	19 (79.2%)	7 (29.2%)	17 (70.8%)	4 (16.7%)	10 (41.7%)	23 (95.8%)	18 (75.0%)	17 (70.8%)	17 (70.8%)	17 (70.8%)
